# Social influence and interaction bias can drive emergent behavioural specialization and modular social networks across systems

**DOI:** 10.1098/rsif.2019.0564

**Published:** 2020-01-08

**Authors:** Christopher K. Tokita, Corina E. Tarnita

**Affiliations:** Department of Ecology and Evolutionary Biology, Princeton University, Princeton, NJ 08544, USA

**Keywords:** self-organization, division of labour, social dynamics, group size, assortativity, homophily

## Abstract

In social systems ranging from ant colonies to human society, behavioural specialization—consistent individual differences in behaviour—is commonplace: individuals can specialize in the tasks they perform (division of labour (DOL)), the political behaviour they exhibit (political polarization) or the non-task behaviours they exhibit (personalities). Across these contexts, behavioural specialization often co-occurs with modular and assortative social networks, such that individuals tend to associate with others that have the same behavioural specialization. This raises the question of whether a common mechanism could drive co-emergent behavioural specialization and social network structure across contexts. To investigate this question, here we extend a model of self-organized DOL to account for social influence and interaction bias among individuals—social dynamics that have been shown to drive political polarization. We find that these same social dynamics can also drive emergent DOL by forming a feedback loop that reinforces behavioural differences between individuals, a feedback loop that is impacted by group size. Moreover, this feedback loop also results in modular and assortative social network structure, whereby individuals associate strongly with those performing the same task. Our findings suggest that DOL and political polarization—two social phenomena not typically considered together—may actually share a common social mechanism. This mechanism may result in social organization in many contexts beyond task performance and political behaviour.

## 1. Introduction

Individuals within social systems differ in their behaviour. When these individual behavioural differences become consistent over time, i.e. when inter-individual behavioural variation exceeds intra-individual behavioural variation, the system is said to exhibit behavioural specialization [1,2]. Under this broad definition, behavioural specialization encompasses well-studied behavioural phenomena, including division of labour (DOL), emergent ‘personalities’ and political polarization. DOL is defined as the specialization of individuals on different tasks—i.e. behaviours necessary for group survival (e.g. foraging)—within the social group. DOL is widespread in both human [3] and animal societies [4] and is associated with beneficial outcomes, such as increased individual reproduction and survival [5]. Personalities—sometimes called behavioural syndromes—are defined as consistent individual differences in non-task behaviours [6–8], such as aggressiveness, exploratory behaviour or cooperation. Diverse personalities have been observed across social systems, from invertebrates [9,10] to birds [11] to mammals [12,13] and, of course, humans. Opinion polarization—an occurrence in human societies—is defined as the adoption of extreme viewpoints by individuals [14]. Opinion polarization can result in political polarization, i.e. specialization of political behaviour, whereby individuals consistently vote for a subset of possible issue stances or political parties. Thus, seemingly distinct behavioural phenomena that are typically studied independently actually fall under the much broader umbrella of behavioural specialization, making it worthwhile to explore possible shared underlying mechanisms for their emergence.

In addition to being widespread across social systems, behavioural specialization often co-occurs with modularity and assortativity in the social network [11,15–21]: both human and animal societies are frequently organized into social networks that show clustering and self-sorting according to behavioural traits, including personality, task specialization or political ideology. In the context of political polarization, the social sciences have theoretically explored the role of social interactions in explaining the co-emergence of behavioural specialization and social network structure [22–25]. In these opinion dynamic models, individuals hold political opinions that can gradually change by becoming similar to the opinions of others with whom they interact. When individuals only interact with those that hold similar opinions—a dynamic known as ‘bounded confidence’—polarization emerges, such that individuals adopt extreme opinions and form ideologically uniform clusters in the social network. While bounded confidence is specific to opinion dynamic models, it is underpinned by two general mechanisms—*social influence* and *interaction bias—*tied in a feedback loop that reinforces behavioural differences in social systems. Social influence refers to the change(s) in individual behaviour in response to the behaviour of social partners. Interaction bias captures individuals' propensity to interact more than by chance with others exhibiting certain traits or behaviours (e.g. homophily [15])*.* Given that political polarization is just a form of behavioural specialization—as are DOL and personalities—it is worth investigating whether a feedback between social influence and interaction bias may underlie the co-emergence of behavioural specialization and social network structure in other contexts and social systems.

Social influence and interaction bias appear to be widespread in animal societies. An animal's social network can shape its behaviour [26–28], suggesting the existence of social influence, whereby individuals change their behavioural traits in response to the behaviour of others. A compelling example comes from social spider groups, where persistent social interactions lead to more varied and consistent personalities over time [29,30], a phenomenon that experiments suggest results from individuals adjusting their personalities to be similar to those with whom they interact [31]. Conversely, an animal's behavioural specialization influences its social connections [11,21,32,33], suggesting the existence of interaction bias, whereby individuals interact more frequently with others exhibiting the same behaviours (e.g. homophily [15]). Interaction bias can result from various factors, such as preferential association among [11,20] or spatial fidelity of [18,34] behavioural types. These two simple mechanisms—social influence and interaction bias—could form a feedback that reinforces behavioural differences in social systems: individuals are more likely to interact with others who are similar, and, via the interaction, their similarity, and therefore the likelihood of future interactions, further increases. Despite the prevalence of these two mechanisms, this possible feedback between them remains underexplored theoretically.

A promising approach to explore this feedback is via the response threshold framework, which has been broadly employed to study collective, self-organized phenomena ranging from DOL to behavioural cascades [25,35–37]. To make specific inferences and predictions, here we focus on the instantiation of this theoretical framework that deals with DOL [5,36,38–40], but this approach is amenable to generalization. DOL is a particularly suitable candidate for two reasons. First, empirical evidence suggests that social interactions can indeed influence task specialization [41–43], but theoretical work so far has only explored social interactions in the context of short-term task recruitment [44–46] or age-based differences in task performance [47,48]. Second, DOL can be employed to study scaling effects [5,38,39,49], i.e. the influence of group size on emergent properties, an aspect that has received less attention in the social science literature despite sociological theory suggesting that group size greatly influences human society [3]. How group size influences social dynamics is especially relevant as the world becomes more global and well connected.

Here we extend the response threshold framework to account for social dynamics—specifically, social influence and interaction bias. We explore both preferential interactions with those who are similar (homophily) and preferential interactions with those who are different (heterophily). We assume interactions to be bidirectional, such that the two participants have the same effect on each other. As a consequence of the interaction, the two individuals may become more similar to each other (henceforth, positive influence) or more different (henceforth, negative influence). We, therefore, consider four possible combinations of interaction bias and social influence: homophily or heterophily with positive or negative influence. Using this framework, we explore the emergence of DOL and the co-emergent social network structure.

## 2. Model description

The response threshold framework relies on two basic assumptions: (a) that there are stimuli signalling information about the environment or the group; and (b) that each individual has internal, stimulus-specific thresholds that determine its behaviour—when a stimulus level exceeds an individual's corresponding threshold, the individual performs the associated behaviour. In the context of DOL, the stimuli are assumed to signal group needs, such as hunger or the need for brood care, and thus the associated behaviours might include foraging or nursing. In the following subsections, we first describe the most common implementation of response thresholds for the study of DOL—the fixed response threshold model, which assumes that an individual's thresholds are fixed (i.e. they do not change over time). Subsequently, we extend this model by relaxing the fixed thresholds assumption to allow an individual's thresholds to change over time due to social influence. We call this the socially modulated threshold model.

### 2.1. Fixed response thresholds

First introduced in the social insect literature [36], fixed response thresholds have been widely used to study self-organized DOL. The dynamics of the model follow the description above: individuals use fixed (i.e. a constant value through time) internal thresholds to respond to stimuli signalling group needs. The model operates in discrete time and assumes a social system with *m* tasks and *n* individuals. Since each threshold corresponds to a specific stimulus, every individual has *m* thresholds. An *n* by *m* binary matrix, *X_t_* = [*x_ij_*_,*t*_], describes the behavioural state of each individual *i* for task *j* at a given time step *t*. We assume individuals can perform at most one task at a time. Therefore, if individual *i* is inactive, all *x_ij_*_,*t*_ = 0; if it is active, exactly one *x_ij_*_,*t*_ = 1.

#### 2.1.1. Stimuli

The model assumes that a given task stimulus is governed by a simple dynamic: it increases when not enough individuals perform the task, and it decreases when sufficiently many individuals perform the task. Specifically, the stimulus for task *j* at time *t*, *s_j_*_,*t*_, satisfies:

$$s_{\;j,t+1}=s_{\;j,t}+\delta_{j}-\alpha \frac{\sum_{i=1}^{n}x_{ij,t}}{n},$$

where *δ_j_* is the constant, task-specific stimulus increase rate per time step for task *j* (i.e. task demand rate), $\sum_{i=1}^{n}x_{ij,t}/n$ is the fraction of the group performing task *j* at time *t*, and *α* is the work efficiency of active individuals. This formulation of the task stimuli dynamics allows task demand to scale proportionally with group size [36].

#### 2.1.2. Thresholds

Only inactive individuals assess stimuli; active individuals cannot switch tasks without first becoming inactive. Every time step, each inactive individual encounters each stimulus in a random order, until it either begins to perform a task and thereby becomes active, or it has encountered all stimuli and thereby remains inactive. When inactive individual *i* encounters a stimulus that exceeds its threshold for that task (*s_j_*_,*t*_ > *θ_ij_*_,*t*_), it performs that task (*x_ij_*_,*t*_ = 1 and all other *x_il_*_,*t*_ = 0 where *l* ≠ *j*). Otherwise, the individual remains inactive for that task. Once an individual becomes active, it continues to perform the task until it spontaneously quits with constant probability *τ* [36,38,39].

Because they are thought to have a biological basis in physiological, genetic or epigenetic differences [50,51] and might therefore have constraints, thresholds are bounded and take values in the interval [0, 100]; however, for completeness, we explore how changing/removing the upper bound on the thresholds affects our results. We initialize all simulations by drawing individual *i*'s threshold for task *j* from a normal distribution with mean *μ_j_* and normalized standard deviation *σ_j_* (measured in units of *μ_j_*; e.g. *σ_j_* = 0.1 indicates a standard deviation that is 10% of the mean). If *σ_j_* = 0, the initial social group is homogeneous with respect to thresholds; if *σ_j_* is positive, then there exists inherent initial variation among group members. For simplicity, we assume that all tasks have the same *σ* and *μ*.

### 2.2. Socially modulated thresholds

To account for social interactions—specifically, social influence and interaction bias—we extend the fixed thresholds model to include inter-individual interactions that can alter the threshold values of individuals, while keeping the stimuli dynamics and the way thresholds determine individual behaviour unchanged. All individuals (both active and inactive) can initiate and be engaged in interactions, but only active individuals exhibit social influence and interaction bias. An *n* by *n* binary matrix *A_t_* = [*a_ik_*_,*t*_], describes the interactions between individuals in a given time step *t*: if individual *i* interacts with individual *k*, then *a_ik_*_,*t*_ = 1; otherwise, *a_ik_*_,*t*_ = 0. For simplicity, we assume that interactions are undirected, i.e. *a_ik_*_,*t*_ = *a_ki_*_,*t*_. To mimic the ephemeral nature of social interactions [52,53], we further assume that an interaction lasts for only one time step.

#### 2.2.1. Social interactions

Every time step, each individual initiates an interaction with exactly one other individual in the group. Nevertheless, an individual can have more than one interaction partner at a time, provided that other individuals initiate interactions with it. When an individual initiates an interaction, a partner is selected from the group probabilistically, using a weighted random sample [54] that represents the random mixing of individuals in the group. The probability that individual *i* selects individual *k* as interaction partner at time *t* is:

$$\mathrm{Pr}(a_{ik,t}=1)=\;\frac{\omega_{ik,t}}{\sum_{k\neq i}\omega_{ik,t}},$$

where *ω_ik_*_,*t*_ is the interaction weight between individuals *i* and *k*. Thus, the probability that individual *i* initiates an interaction with individual *k* is proportional to interaction weight *ω_ik_*_,*t*_. The interaction weights are determined by the interaction bias.

#### 2.2.2. Interaction bias

Inactive individuals exhibit no interaction bias (i.e. they are just as likely to initiate an interaction with active and inactive individuals). Active individuals can exhibit interaction bias towards other active individuals who perform the same task. When individuals tend to positively bias their interactions towards those performing the same behaviour, they are said to exhibit homophily [15]; conversely, a positive bias towards those performing different behaviours is called heterophily. To allow for interaction bias, we let the interaction weights assigned to potential partners depend on their behavioural state. If individual *k* is performing the same task as individual *i* at time *t*, individual *k* is assigned interaction weight *ω_ik_*_,*t*_ = *β* ≥ 0; otherwise, if individual *k* is inactive or performing a different task, then it is assigned interaction weight *ω_ik_*_,*t*_ = 1. If *β* > 1, then the model captures homophily; if *β* < 1, it captures heterophily. Setting *β* = 1 causes interactions to be entirely random, such that groups are well mixed and the probability of individual *i* interacting with individual *k* is 1/(*n* − 1). Inactive individuals have an equal chance of interacting with every behavioural type (i.e. all *ω_ik_*_,*t*_ = 1). The interaction bias could be driven by preferential association between behavioural types, but also by spatial factors alone. For example, the spatial segregation of tasks may limit the individuals with whom an active individual can interact: when nursing larvae in a location, an individual is more likely to bump into other nurses and less likely to bump into foragers that are away from the nest searching for food. Therefore, interaction bias *β* could be thought of as a spatially implicit term, depending on the system under study.

#### 2.2.3. Social influence

Thresholds change as a result of social interactions with active individuals. When two inactive individuals interact, all their thresholds remain unchanged. Active individuals influence the thresholds of those they interact with (both active and inactive). Specifically, when individual *i* interacts with an active individual performing task *j*, individual *i*'s threshold for task *j* decreases by *ε*, while its other thresholds increase by the same amount. If *ε* > 0, then this makes individual *i* more likely to perform the same task as the interaction partner in the future (*positive influence*); if *ε* < 0, individual *i* is less likely to perform the same task (*negative influence*). Because interactions are undirected, both interacting individuals will change each other's thresholds, provided that both are actively performing tasks. Thus, the modification of individual *i*'s thresholds is dependent on the number of active individuals it interacts with and can be described by:

$$\theta_{ij,t+1}=\theta_{ij,t}+\varepsilon \left(\underset{k\neq i,l\neq j}{\sum }a_{ik,t}x_{kl,t}-\underset{k\neq i}{\sum }a_{ik,t}x_{kj,t}\right),$$

where $\sum a_{ik,t}x_{kl,t}$ is the number of active interaction partners performing tasks other than task *j* and $\sum a_{ik,t}x_{kj,t}$ is the number of active interaction partners performing task *j*. It is important to note that when *ε* = 0, thresholds are fixed, although social interactions still occur. Thus, setting *ε* = 0 captures the fixed threshold model described in section 2.1, but with the addition of social interactions (that do not influence the emergent self-organization). This will allow us to both compare the socially modulated results with the fixed threshold model and explore the emergent social network structure in the absence of social influence.

All parameters and their values can be found in table 1.

**Table 1.** Parameter settings for model. Unless stated otherwise, these are the default values used in all simulations.

parameters	description	values or range in simulation
*T*	simulation length in time steps	50 000
*n*	number of individuals	5–100
*m*	number of tasks	2
*μ_j_* = *μ*	task-specific mean of initial distribution of thresholds for task *j*; taken to be the same for all tasks	50
*σ_j_* = *σ*	task-specific initial threshold variation (*by model type*); taken to be the same for all tasks	0 (socially modulated), 0.05 (fixed)
*δ_j_* = *δ*	rate of stimulus increase for task *j*; taken to be the same for all tasks	0.8
*α*	work efficiency of active individuals	2
*τ*	probability of quitting task once active	0.2
*ε*	social influence (*by model type*)	±0.1 (socially modulated), 0 (fixed)
*β*	interaction bias	1.1 (homophily), 0.9 (heterophily)

## 3. Results

For simplicity, we assumed that there are only two tasks, i.e. *m* = 2, that both tasks have the same demand rate *δ*, and that thresholds are drawn from the same distribution, with mean *μ* and relative standard deviation *σ*. To investigate emergent DOL and the co-emergent social network structure in the presence of social influence and interaction bias, we started with a homogeneous population (i.e. *σ* = 0). Subsequently, to determine whether the co-emergent social network structure requires *both* social influence and interaction bias (as opposed to interaction bias alone), we compared our results to those of a model in which individuals still interacted in a biased manner but had fixed thresholds (i.e. *ε* = 0). For this model, since thresholds are fixed, in order for DOL to emerge and permit comparison, we started with a slightly heterogeneous population (i.e. *σ* is small, but non-zero).

### 3.1. Emergent DOL

**Figure 1.**
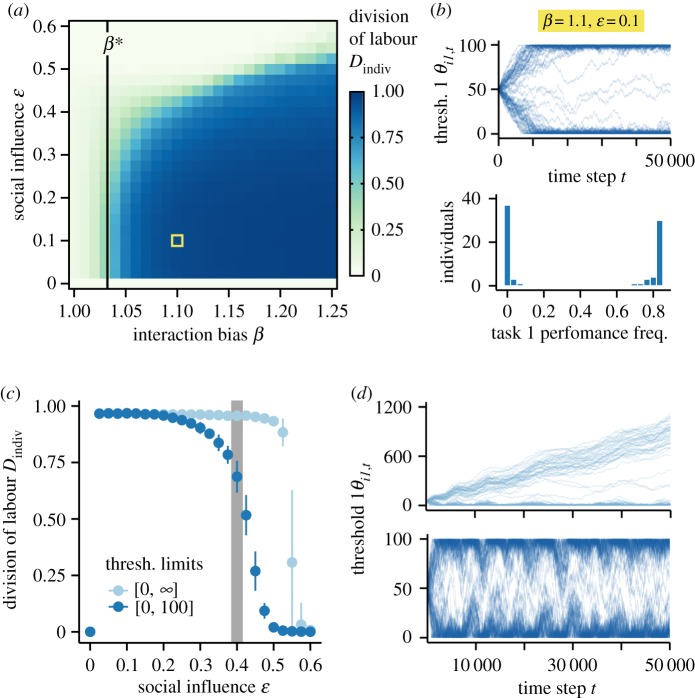
The emergence of behavioural specialization under homophily with positive influence. Simulations and calculations assume group size *n* = 80. (*a*) The effect of interaction bias *β* and social influence *ε* on the emergence of behavioural specialization. Colour represents the mean of 100 replicate simulations for each parameter combination. The black line represents the analytical solution for the minimal level of interaction bias, β*, needed for the emergence of DOL. (*b*) Example simulation from the highlighted parameter combination in (*a*), showing the change in individuals' task 1 thresholds over time and the cumulative distribution of task 1 performance frequency. (*c*) The effect of threshold bounds on suppressing DOL at high levels of social influence *ε*. Points represent mean (±s.d.) of 100 replicates. (*d*) Example simulations from parameter values highlighted in grey in (*c*), showing the change in individuals' task 1 threshold over time. (Online version in colour.)

We found that homophily with positive influence—the combination that is often implemented in opinion dynamic models—could result in self-organized DOL (figure 1*a*). Under these conditions, social influence and interaction bias formed a positive feedback loop that caused thresholds to polarize, such that individuals had one very low and one very high threshold (figure 1*b*). Since one stimulus always crossed a given individual's low threshold and the other stimulus never crossed its high threshold, individuals fully specialized in one task. However, this feedback loop resulting in DOL could not form when either interaction bias was insufficient or social influence was absent. Analytical results revealed that a minimum level of interaction bias β* must be present in order for active individuals to have their behaviour reinforced by others performing the same task, and thus for DOL to emerge. This outcome is due to the fact that an active individual cannot interact with itself (electronic supplementary material). Although the numbers of individuals performing each task at a given time are similar (i.e. *n*_1_ ≈ *n*_2_), an individual performing task 1 has *n*_1_ − 1 potential interaction partners performing the same task and *n*_2_ potential interaction partners performing the other task. Therefore, the interaction bias *β* must be large enough to overcome the deficit in potential interaction partners, i.e. *β*(*n*_1_ − 1) > *n*_2_. When there are more than two tasks, there is an even larger deficit in potential interaction partners, which requires even higher *β* in order to achieve (high levels of) DOL (electronic supplementary material, figure S2A). The order in which inactive individuals encounter stimuli has no effect when there are two tasks but can have an effect when there are more than two tasks (electronic supplementary material, figure S2B–D).

On the other hand, if the social influence was not present, there was no mechanism by which the initially homogeneous population could differentiate and, therefore, DOL could not emerge. Interestingly, while the presence of positive influence generally resulted in DOL, high levels of positive influence could actually suppress DOL (figure 1*a,c*) by causing individuals to become overly similar to one another (i.e. we observed the emergence of conformity). This conformity may be due to the fact that high social influence causes thresholds to change at a faster rate than stimuli can regenerate. As a result, individuals become more influenced by each other's behaviour than by the demand for various tasks, even when there are far more individuals performing one task than the other (and therefore much less demand for the former task). This eventually leads all individuals to perform the already more-performed task. In the simulations, removing upper limits on threshold values caused this conformity to occur at higher levels of social influence compared to when there are bounds on threshold values (figure 1*c*). This was due to the fact that threshold bounds keep the two threshold values of each individual close enough that, when there are temporarily more individuals performing one task than the other, if the social influence is sufficiently high, it takes only a few interactions to decrease the threshold bias of individuals specialized on the less-performed task and thereby reduce behavioural specialization (figure 1*d*); however, when thresholds are unbounded, the difference between an individual's two thresholds can grow so large that a temporary shift in threshold values is not enough to meaningfully alter its behaviour, unless the social influence is very high.

**Figure 2.**
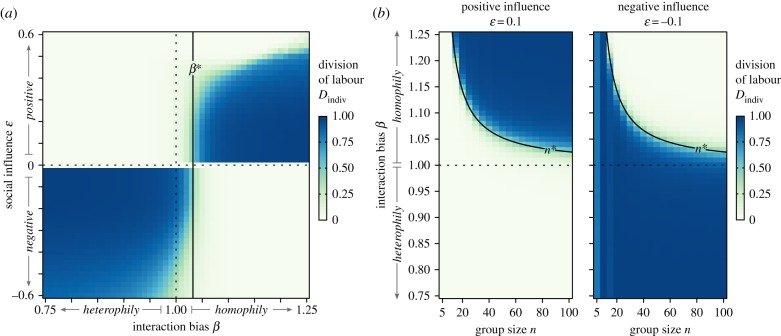
Emergent behavioural specialization for different types of social interactions and as a function of group size. Colour represents the mean of 100 replicate simulations for each parameter combination. (*a*) DOL in groups of size *n* = 80 under different combinations of social influence and interaction bias. Black line is the analytical calculation for β*, the minimal level of interaction bias at which active individuals are more likely to interact with others performing the same task. (*b*) The effect of group size and interaction bias on DOL under either positive or negative influence. Black curve on plot is the analytical calculation of n*, the minimal group size at which active individuals become more likely to interact with others performing the same task. Such a switch in interaction patterns with group size only occurs with homophily; with heterophily, active individuals are always more likely to interact with individuals performing the other task. (Online version in colour.)

To explore other social interaction types and their effect on social organization, we simulated three additional combinations of social influence and interaction bias—*homophily with negative influence*; *heterophily with positive influence;* and *heterophily with negative influence* (figure 2*a*). Each combination resulted in a unique pattern of emergent DOL, but a general trend can be observed: interaction bias only affected emergent DOL under conditions of homophily. Increasing homophily in the presence of positive influence resulted in a shift from homogeneous, non-specialized individual behaviour to highly specialized individual behaviour, while increasing homophily in the presence of negative influence resulted in the opposite trend, i.e. a shift from specialized to non-specialized behaviour. On the contrary, under conditions of heterophily, the level of interaction bias did not affect the pattern of behavioural specialization: regardless of the intensity of heterophily, DOL always emerged when heterophily was combined with negative influence, whereas DOL never emerged—and groups remained behaviourally homogeneous—when heterophily was combined with positive influence. Lastly, high levels of social influence still decreased DOL, except under heterophily with positive influence, where groups were always homogeneous in behaviour.

Group size influenced DOL, but only under homophily. Homophily with positive influence caused DOL to increase with group size, while homophily with negative influence caused DOL to decrease with group size (figure 2*b*; electronic supplementary material, figure S3). Under heterophily, however, group size did not affect DOL: heterophily with positive influence resulted in homogeneous, non-specialized behaviour at every group size, while heterophily with negative influence resulted in fully specialized behaviour at every group size. Analytical results showed that, under our assumptions, scaling effects could never occur with heterophily because an active individual is always more likely to interact with individuals performing the other task, regardless of the group size (electronic supplementary material). Under homophily, however, there are groups large enough so that an active individual is more likely to interact with those performing the same task. For a given interaction bias *β* > 1, there is a minimal group size n* = *αβ*/*δ*(*β* − 1) that allows the interaction bias to overcome the deficit in potential interaction partners (electronic supplementary material). For groups of size larger than n*, increasing group size enhanced the effect of interaction bias *β*, such that individuals increasingly interacted with those performing the same task. The higher frequency of interaction among similar individuals caused thresholds to change at a faster rate and eventually polarize, such that all individuals' thresholds became maximally biased towards one task or the other (electronic supplementary material, figure S4). Thus, the feedback between social influence and interaction bias was amplified in larger groups, which, in turn, caused DOL to emerge faster over the course of a simulation. Moreover, the level of interaction bias β* needed for DOL to emerge decreased as group size increased (electronic supplementary material, figure S5).

Although, for computational convenience, we ran simulations on short-to-intermediate timescales, longer simulations revealed that the results do not qualitatively change between shorter and longer timescales (electronic supplementary material, figure S6) and analytical calculations produced further confirmation (electronic supplementary material).

### 3.2. Co-emergent social network structure

**Figure 3.**
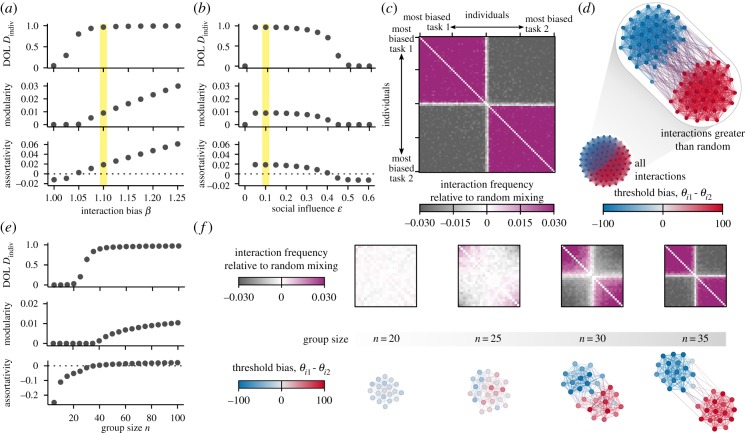
Socially modulated thresholds result in the co-emergence of behavioural specialization and modular, assortative social networks. Simulations in (*a*–*d*) use group size *n* = 80. The effect of (*a*) interaction bias and (*b*) social influence on DOL, social network modularity, and social network assortativity. Points represent mean (±s.d.) of 100 replicates. (*c*) The average interaction matrix representing all 100 replicate simulations of the specific parameter combination highlighted in (*a,b*) (*β* = 1.1, *ε* = 0.1). Each entry represents the interaction frequency between the individual on the row and the individual on the column. Individuals in the matrix are sorted according to threshold bias, such that the individuals most biased towards task 1 are in the upper left and the individuals most biased towards task 2 are in the bottom right. (*d*) Example social network from one simulation, shown in two renditions: bottom rendition shows all interactions; top rendition shows only interactions that occurred more frequently than what would be expected by random mixing. (*e*) The effect of group size on DOL, social network modularity and social network assortativity. (*f*) Average interaction matrices and example social networks—showing only interactions more frequent than random mixing—for the range of group sizes corresponding with the emergence of DOL. (Online version in colour.)

Since homophily with positive influence offered the most interesting case for emergent DOL that scales positively with group size, we focused the remainder of our analysis on this subset of social interactions. At a given group size, for combinations of social influence and interaction bias that allowed the emergence of DOL, the co-emergent social network was fully connected, but the frequency of interactions between individuals showed an assortative, modular pattern. The modularity and assortativity of the network increased with the interaction bias *β*, i.e. the social network structure increasingly deviated from random (figure 3*a*). On the other hand, the social influence, *ε*, did not generally affect the social network structure, except at high levels of positive influence, where modularity and assortativity decreased and eventually disappeared (figure 3*b*). In instances where both social influence and interaction bias were present and DOL emerged, the most frequent interactions occurred within clusters of individuals with similar threshold biases (figure 3*c*,*d*). The frequency of interactions within these clusters was significantly higher than random (see Material and methods for explanation of statistical test), while the frequency of interactions between individuals in different clusters was significantly lower than random. As with DOL, group size also influenced social network structure (figure 3*e*). As groups increased in size, both modularity and assortativity rapidly emerged over a narrow range of group sizes coinciding with the emergence of DOL (figure 3*f*).

### 3.3. Social influence is necessary for modularity to emerge

**Figure 4.**
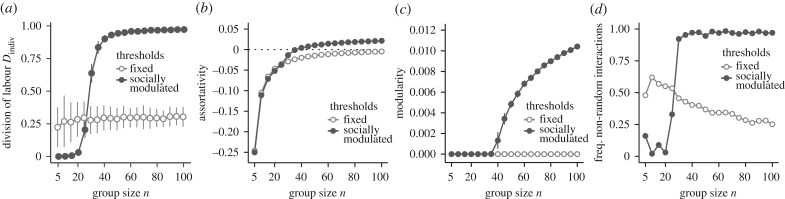
Social influence is needed for scaling effects to arise. In (*a*–*c*), points represent mean (±s.d.) of 100 replicates. Comparing scaling effects of (*a*) behavioural specialization, (*b)* assortativity, (*c*) modularity and (*d*) frequency of non-random interactions between socially modulated thresholds and fixed thresholds.

Finally, we investigated whether the observed social network structure was the result of *both* social influence and interaction bias or was simply the result of interaction bias alone. To this end, we compared our results to the output of a model in which individuals still interacted in a biased manner but had fixed thresholds (i.e. *ε* = 0). In our socially modulated threshold model, threshold variation is generated over time due to social influence, even in initially homogeneous groups; however, in response to threshold models, DOL will emerge only if there is initial variation in thresholds among individuals. Therefore, to allow comparison, we ran fixed threshold simulations with initial threshold variation *σ* = 0.05, which was sufficient to reach high DOL (figure 4*a*; electronic supplementary material, figure S7).

When we removed the effects of social influence on thresholds using the fixed threshold model, we found that interaction bias alone resulted in different social network patterns. First, while both fixed and socially modulated thresholds resulted in increasing assortativity with group size, the increase in assortativity was larger and eventually positive with socially modulated thresholds (figure 4*b*), whereas, with fixed thresholds, assortativity plateaued at approximately random mixing among individuals. Second, socially modulated thresholds resulted in increasingly modular social networks with group size, while fixed thresholds resulted in consistently non-modular social networks (figure 4*c*). Finally, as groups increased in size, socially modulated thresholds resulted in more non-random interactions (material and methods); on the contrary, fixed thresholds resulted in fewer non-random interactions as groups increased in size (figure 4*d*). Examining individual-level behaviour revealed that these contrasting patterns of social network structure were due to task generalists (electronic supplementary material, figure S8). Because fixed thresholds do not change and therefore remain in their initial normal distribution, a sizeable number of individuals have thresholds from the middle of this distribution and are therefore equally likely to do either task. As such, these ‘generalists’ interact frequently with both types of task specialists, thereby bridging the social network and preventing the establishment of modularity. As groups grew larger, the number of generalists increased, which in turn increased the frequency of random interactions in the social network. By contrast, socially modulated thresholds caused individuals to have strongly biased thresholds at larger group sizes, thereby removing any initial generalists from the group. Taken together, these results demonstrate that both social influence and interaction bias are necessary to produce the scaling effects in social network structure that were observed in the simulations.

## 4. Discussion

Our main result demonstrates that, in the presence of homophily with positive influence, the feedback between social influence and interaction bias could result in the co-emergence of DOL and modular social network structure. These results reveal that self-organized specialization could give rise to modular social networks without direct selection for modularity, filling a gap in our knowledge of social organization [55] and mirroring findings in gene regulatory networks, which can become modular as genes specialize [56]. The co-emergence requires both social influence and interaction bias but, if the level of social influence is too high, its pressure leads to conformity, which homogenizes the society. Because this feedback between social influence and interaction bias has also been shown to drive political polarization [22–25], our results suggest a shared mechanism between two social phenomena—polarization and DOL—that have not traditionally been considered together and raise the possibility that this mechanism may structure social systems in other contexts as well, such as in the case of emergent personalities [11,29–31]. Furthermore, the ubiquity of this mechanism may help explain why social systems often have a common feature—modular network structure—that is shared with a range of other biological and physical complex systems [57].

Intriguingly, although our results suggest that diverse forms of behavioural specialization—and the associated modular, assortative social networks—might arise from a common mechanism, depending on their manifestation, they can be either beneficial or detrimental for the group. For example, DOL and personality differences have long been associated with beneficial group outcomes in both animal [5,58–60] and human societies [61] (although it can sometimes come at the expense of group flexibility [62]). Moreover, the modularity that co-occurs in these systems is also often framed as beneficial, since it can limit the spread of disease [63] and make the social system more robust to perturbation [55]. On the contrary, political polarization is typically deemed harmful to democratic societies [64]. Thus, an interesting question for future research arises: if a common mechanism underlies the emergence of behavioural specialization and the co-emergence of a modular social network structure in multiple contexts, why would group outcomes differ so dramatically? Insights may come from studying the frequency of co-occurrence among various forms of behavioural specialization. If the same mechanism underlies behavioural specialization broadly, then one would expect multiple types of behavioural specialization (e.g. in task performance, personality, decision-making) to simultaneously arise and co-occur in the same group or society, as is the case in some social systems, where certain personalities consistently specialize on particular tasks [9,10] or in human society, where personality type and political ideology appear correlated [65]. Then, the true outcome of behavioural specialization for the group is the net across the different types co-originating from the same mechanism and cannot be inferred by investigating any one specific instantiation of behavioural specialization.

While DOL emerged when homophily was combined with positive influence, other combinations of social influence and interaction bias may nevertheless be employed in societies to elicit other group-level phenomena. For instance, under certain conditions, a society might benefit from uniform rather than divergent, specialized behaviour. This is the case when social insect colonies must relocate to a new nest, a collective decision that requires consensus-building [66]. To produce consensus, interactions should cause individuals to weaken their commitment to an option until a large majority agrees on one location. Heterophily with positive influence—preferential interactions between dissimilar individuals that reduce dissimilarity—achieves this dynamic and is consistent with the cross-inhibitory interactions observed in nest-searching honeybee swarms [67]: scouts interact with scouts favouring other sites and release a signal that causes them to stop reporting that site to others. One could imagine that similar dynamics might also reduce political polarization.

Recent work has shown that built environments—physical or digital—can greatly influence collective behaviour [16,18,68–70], but the mechanisms underlying this influence have remained elusive. By demonstrating the critical role of interaction bias for behavioural outcomes, our results provide a candidate mechanism: structures can enhance interaction bias among individuals and thereby amplify the behavioural specialization of individuals. For example, nest architecture in social insect colonies alter collective behaviour [68] and social organization [18] possibly because the nest chambers and tunnels force proximity to individuals performing the same behaviour and limit interactions with individuals performing other behaviours. Similarly, the Internet and social media platforms have changed the way individuals interact according to interest or ideology [16,69,70]: selective exposure to certain individuals or viewpoints creates a form of interaction bias that our results predict would increase behavioural specialization, i.e. political bias. Thus, our model predicts that built environments should increase behavioural specialization beyond what would be expected in more ‘open’, well-mixed environments. This prediction has evolutionary consequences: a nest can increase behavioural specialization without any underlying genetic or otherwise inherent, diversity. Such consequences would further consolidate the importance of built environments—specifically, nests—for the evolution of complex societies. It has been previously argued that the construction of a nest may have been a critical step in the evolution of stable, highly cooperative social groups [71]. Subsequent spatial structuring of the nest would then, according to our findings, bring further benefits to nascent social groups in the form of increased behavioural specialization, e.g. DOL, even in the absence of initial behavioural and/or trait heterogeneity.

Finally, our results shed light on how plastic traits can result in scaling effects of social organization with group size, a finding that tightens theoretical links between the biological and social sciences. Founding sociological theorist, Emile Durkheim, posited that the size of a society would shape its fundamental organization [3]: small societies would have relatively homogeneous behaviour among individuals, but DOL would naturally emerge as societies grew in size and individuals differentiated in behaviour due to social interactions. Similar to Durkheim's theoretical framing, John Bonner famously posited that complexity, as measured by the differentiated types of individuals (in societies) or cells (in multicellular aggregations), would increase as groups grew in size [72]. Bonner argued that the differentiation among individuals was not due to direct genetic determinism but was instead the result of plasticity that allowed individuals to differ as groups increased in size. Our model supports these qualitative predictions and even predicts a rapid transition in organization as a function of group size that results from socially influenced plasticity at the level of the individual. Previous theoretical work showed that DOL could exhibit group size scaling effects even with fixed traits, but these increases in DOL quickly plateaued past relatively small group sizes [5,39]. Our model, along with models of self-reinforced traits [38], demonstrates how DOL could continue to increase at larger group sizes, a pattern observed empirically in both animal [49,73] and human societies [74,75]. For other forms of behavioural specialization, such as emergent personalities or political polarization, the effect of group size is understudied; however, our results suggest similar patterns. Our model further demonstrated that group size can affect social network structure, a dynamic that has only been preliminarily investigated empirically so far [76]. Leveraging new technologies—such as camera-tracking algorithms and social media—that can simultaneously monitor thousands of individuals and their interactions to investigate the effect of group size on societal dynamics could have significant implications as globalization, urbanization and technology increase the size of our social groups and the frequency of our interactions.

## 5. Material and methods

### 5.1. Simulation details

We implemented our computational model as an agent-based model and ran 100 replicate simulations per parameter combination. Each simulation lasted *T* = 50 000 time steps.

### 5.2. Measuring behavioural specialization and DOL

DOL is measured with an information theory metric that is common in social insect research [1]. At the end of a simulation (*t* = *T*), a behavioural matrix is constructed with individuals as rows and tasks as columns, i.e. *X* = [*x_ij_*]. Each entry $x_{ij}=\sum_{t=1}^{T}x_{ij,t}$ describes the amount of time that individual *i* spent performing task *j* over the course of the simulation. Using this matrix, we then calculated the *D*_indiv_ metric, which measures the degree to which individuals specialize on a single task. *D*_indiv_ takes values *D*_indiv_ ∈ [0, 1], such that 0 signifies homogeneous behaviour and 1 signifies complete DOL.

### 5.3. Analysing social network structure

We construct social networks by aggregating all interactions over the course of the simulation (electronic supplementary material, figure S1A), a common approach for time-ordered networks [52,53]. Thus, each simulation yields an interaction matrix *A* = [*a_ik_*], whose entries represent the interaction frequency between individuals *i* and *k* over the course of the simulation, i.e. $a_{ik}=\sum_{t=1}^{T}a_{ik,t}/T$.

We analysed the network structure by sorting the interaction matrix according to threshold bias, calculated using each individual *i*'s final threshold values: *θ_i_*_1,*t*=_*_T_* − *θ_i_*_2,*t*=_*_T_*. This value quantifies an individual's final internal bias towards one task or the other and takes values in the range [− 100, 100], whereby −100 and 100 signify maximum bias towards task 1 and task 2, respectively. Individuals within a group were then ranked according to threshold bias, such that the individual most biased towards task 1 was assigned rank 1 and the individual most biased towards task 2 was assigned rank *n* (electronic supplementary material, figure S1B). This ranking and sorting was done for all simulations of a given group size. To then calculate an average social network describing the typical interaction patterns within a group of a given size, we averaged over all sorted interaction matrices for that group size (electronic supplementary material, figure S1C). The resulting average interaction matrix describes interactions among individuals in relative terms—e.g. on average how did the individual most biased towards task 1 interact with others in the group. In our figures throughout, matrices are the average of 100 replicate simulations of a given parameter combination, while networks are an example from a single simulation.

*Modularity* is a form of community structure within a group in which there are clusters of strongly connected nodes that are weakly connected to nodes in other clusters. Using each simulation's time-aggregated interaction matrix *A*, we calculated modularity with the metric developed by Clauset *et al.* [77]. A modularity value of 0 indicates that the network is a random graph and, therefore, lacks modularity; positive values indicate deviations from randomness and the presence of some degree of modularity in the network.

*Frequency of non-random interactions* reveals the degree to which individuals are biasing their interactions towards or away from certain other individuals. For a random, well-mixed population, the expected frequency of interactions between any two individuals is *p*_interact_ = 1 − (1 − 1/(*n* − 1))^2^. For our resulting social networks, we compared this expected well-mixed frequency to the value of each entry *a_ik_* in the average interaction matrix resulting from the 100 replicate simulations per group size. To determine whether the deviation from random was statistically significant, we calculated the 95% confidence interval for the value of each entry *a_ik_* in the average interaction matrix. If the 95% confidence interval for a given interaction did not cross the value *p*_interact_, that interaction was considered significantly different than random.

*Assortativity* is the tendency of nodes to attach to other nodes that are similar in some trait (e.g. here, threshold bias). We measured assortativity using the weighted assortment coefficient [78]. This metric takes values in the range [− 1, 1], with positive values indicating a tendency to interact with individuals that are similar in traits and negative values indicating a tendency to interact with individuals that are different. A value of 0 means random traits-based mixing among individuals.

## Supplementary Material

Supplemental text and figures

## Data accessibility

All code used to simulate the model and analyse model outputs, as well as copies of the simulation data shown in this paper, can be found on GitHub at https://github.com/christokita/socially-modulated-threshold-model.
