# Supplementary material for: Social influence and interaction bias can drive emergent behavioural specialization and modular social networks across systems
Source: J R Soc Interface. 2020 Jan 8;17(162):20190564. doi: 10.1098/rsif.2019.0564 (PMC7014790; doi:10.1098/rsif.2019.0564)
Supplement: Supplemental text and figures [file rsif20190564supp1.pdf]

Supplementary Information for:
Social influence and interaction bias can drive emergent behavioural specialization and modular social networks across systems
Journal of the Royal Society Interface

Christopher K. Tokita[1*], Corina E. Tarnita[1],

1 Department of Ecology and Evolutionary Biology, Princeton University, Princeton, NJ 08544, USA

*** ctokita@princeton.edu**

Supplemental text

Here, we perform a simple and approximate analytical exploration of the effect of interaction bias β and group size n on behavioural specialization in the socially modulated threshold model. We consider the same scenario explored in our simulations: there are two tasks ($m = 2$) and they have the same demand ($\delta_1 = \delta_2 = \delta$). Provided that the task demand is not too high relative to the work performance efficiency of individuals (i.e. δ/α is not too high), and that the social influence ε is not so high that differences in an individual's two thresholds get amplified quickly, then the simulations reveal that the stimuli levels reach a quasi-steady state (it is a stochastic system, so fluctuations continue to occur around a mean). In other words, the workers are able to meet the work demands. Henceforth, for the purpose of this calculation, we will ignore the fluctuations. Despite this simplifying assumption, our results are in very good agreement with the simulations (see Fig. S5).

Since the change in stimulus level for task j is given by

$$s_{j,t+1} - s_{j,t} = \delta - \alpha\frac{n_{j,t}}{n} \tag{1}$$

where $n_{j,t}$ is the number of individuals performing task j at time t, at steady state $s_{j,t+1} - s_{j,t} = 0$ yields:

$$n_{j,t} = \frac{\delta}{\alpha}n \tag{2}$$

Thus, at steady state both tasks are expected to be performed by the same number of individuals, i.e. $n_{1,t} = n_{2,t}$.

Next, we take a focal individual i that is performing task 1 at time t and we consider the change in its threshold values. For this calculation we assume that threshold values are unbounded, so the two thresholds do not reach a steady state even if the stimulus level does. We can describe the expected change in individual i's two thresholds in one time step as:

$$\theta_{i1,t+1} - \theta_{i1,t} = -\varepsilon E_{11,t} + \varepsilon E_{12,t} \tag{3}$$

$$\theta_{i2,t+1} - \theta_{i2,t} = \varepsilon E_{11,t} - \varepsilon E_{12,t} \tag{4}$$

where ε is the social influence parameter and $E_{11,t}$ (respectively, $E_{12,t}$) is the expected number of interaction partners performing task 1 (respectively, task 2) at time t.

To determine the number of expected interaction partners, $E_{11,t}$ and $E_{12,t}$, note that individuals cannot interact with themselves. As a result, if individual i is performing task 1 at time t, then there are actually $n_{1,t} - 1$ potential partners performing task 1 and $n_{2,t}$ potential partners performing task 2 (inactive individuals do not exert social influence and therefore accounting for their interactions is not necessary when calculating expected threshold change). Thus:

$$E_{11,t} = \frac{\beta(n_{1,t}-1)}{\beta(n_{1,t}-1)+(n-n_{1,t})} + (n_{1,t}-1)\frac{\beta}{\beta(n_{1,t}-1)+(n-n_{1,t})} - \frac{\beta^2(n_{1,t}-1)}{(\beta(n_{1,t}-1)+(n-n_{1,t}))^2} \tag{5}$$

where the left-most term is the expected number of interactions the focal individual will initiate with an individual performing task 1, the middle term is the expected number of individuals performing task 1 that will initiate an interaction with the focal individual, and the right-most term is the chance that the focal individual initiates an interaction with an individual who also happens to initiate an interaction with the focal (which still counts as only one interaction).

Following the same logic as above, the expected number of individuals performing task 2 that individual i will interact with is:

$$E_{12,t} = \frac{n_{2,t}}{\beta(n_{1,t}-1)+(n-n_{1,t})} + n_{2,t}\frac{1}{\beta(n_{2,t}-1)+(n-n_{2,t})} - \frac{n_{2,t}}{(\beta(n_{1,t}-1)+(n-n_{1,t}))^2} \tag{6}$$

Positive influence. If we assume positive influence, i.e., $\varepsilon > 0$, then threshold θ_{i1} is expected to decrease—and threshold θ_{i2} is expected to increase—if $E_{11,t} > E_{12,t}$. Over time, this would result in individual i fully specializing in task 1. Thus, a positive feedback loop between interaction bias and positive influence is only expected to emerge if individuals performing a task are more likely, on average, to interact with those performing the same task.

We can then use this inequality to solve for the group size n^* and interaction bias β^* at which behavioural specialization would emerge. The full solution can only be obtained numerically and does not have a compact form. This is due to the quadratic terms in equations (5) and (6) that ensure that we do not double count interactions; however, these terms have only a small influence on the final result (see Fig. A) because the likelihood of two individuals picking each other simultaneously is very small. Ignoring these quadratic terms provides a simple, intuitive solution that is very close to the full numerical one.

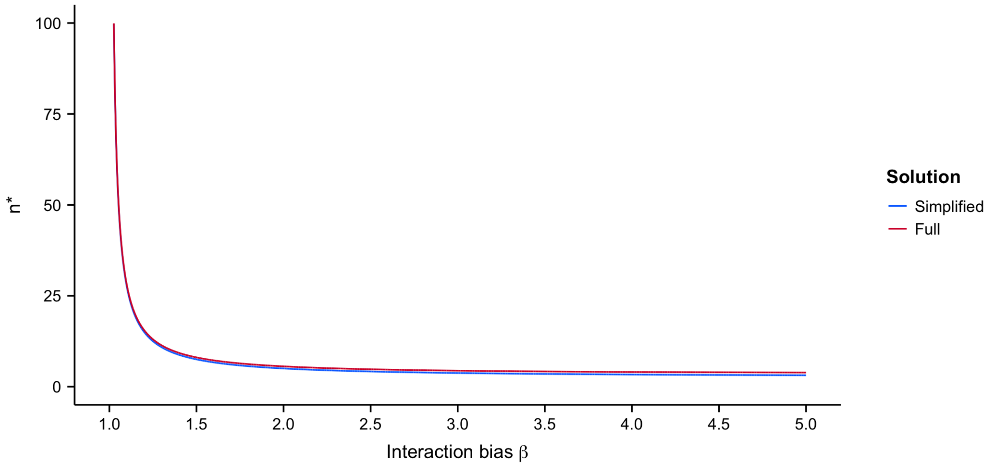

Figure A. Difference in solution for n^* when including or excluding the quadratic term.

With this simplification, and given that $n_{1,t} = n_{2,t}$ at steady state, $E_{11,t} > E_{12,t}$ becomes:

$$\beta(n_{1,t}-1) > n_{2,t} \quad \Longrightarrow \quad \beta\left(\frac{\delta}{\alpha}n - 1\right) > \frac{\delta}{\alpha}n \tag{7}$$

Note that, under these conditions, inequality (7) can never be true if $\beta \leq 1$. This reveals why, when tasks are equally demanding, scaling effects emerge only under homophily. If there is no bias towards individuals performing the same behaviour ($\beta \leq 1$), then there is never a group large enough that an individual will be more likely to encounter those performing the same behaviour.

We can now use inequality (7) to see how group size influences the emergence of behavioural specialization. Assuming $\beta > 1$, we can solve for group size n to determine the critical group size n^* at which behavioural specialization will emerge:

$$n^* = \frac{\alpha\beta}{\delta(\beta-1)} \tag{8}$$

We can also rearrange this equation to solve for the critical interaction bias β^* at which behavioural specialization should emerge:

$$\beta^* = \frac{n\delta}{\delta n - \alpha} \tag{9}$$

Equation (8) reveals a few interesting dependencies. First, increasing interaction bias (assuming $\beta > 1$) decreases the group size at which behavioural specialization will emerge. One could then think of interaction bias β as making the emergence of behavioural specialization "easier" at a given group size. As β increases, the probability of interacting with those performing the same behaviour will increase relative to the probability of interacting with individuals performing other behaviours. If β increases beyond β^*, it will then increase the

speed at which the thresholds $\theta_{ij,t}$ change. On the other hand, we see that increasing α—task performance efficiency, or broadly, the rate at which individual behaviour reduces the stimulus associated with task need—increases the group size at which behavioural specialization will emerge. This is because the more efficient active individuals are, the fewer are needed to perform a task, which then decreases the amount of behavioural reinforcement for active individuals. In our model, we make efficiency α scale proportionally to the number of tasks m, i.e., $\alpha = m$. Under this assumption, increasing m—the number of possible tasks, behaviours, or political parties—could increase the group size at which behavioural specialization will emerge. Lastly, we see that increasing the stimulus regeneration rate δ, i.e., the task demand rate, will decrease the group size at which behavioural specialization emerges. This is because δ increases the number of individuals needed to perform a task, meaning there is more behavioural reinforcement for active individuals.

Negative influence. We can perform a similar analysis for negative social influence, where the condition that needs to be met is: $-\varepsilon E_{11,t} + \varepsilon E_{12,t} < 0$.

Summary. Overall, we can summarize our results as follows:

Social influence	Interaction Bias	Condition for full specialization
Positive ($\varepsilon > 0$)	Homophily ($\beta > 1$)	$n > \frac{\alpha\beta}{\delta(\beta-1)}$
Negative ($\varepsilon < 0$)	Homophily ($\beta > 1$)	$n < \frac{\alpha\beta}{\delta(\beta-1)}$
Positive ($\varepsilon > 0$)	Heterophily ($\beta < 1$)	Never
Negative ($\varepsilon < 0$)	Heterophily ($\beta < 1$)	Always

Supplemental figures

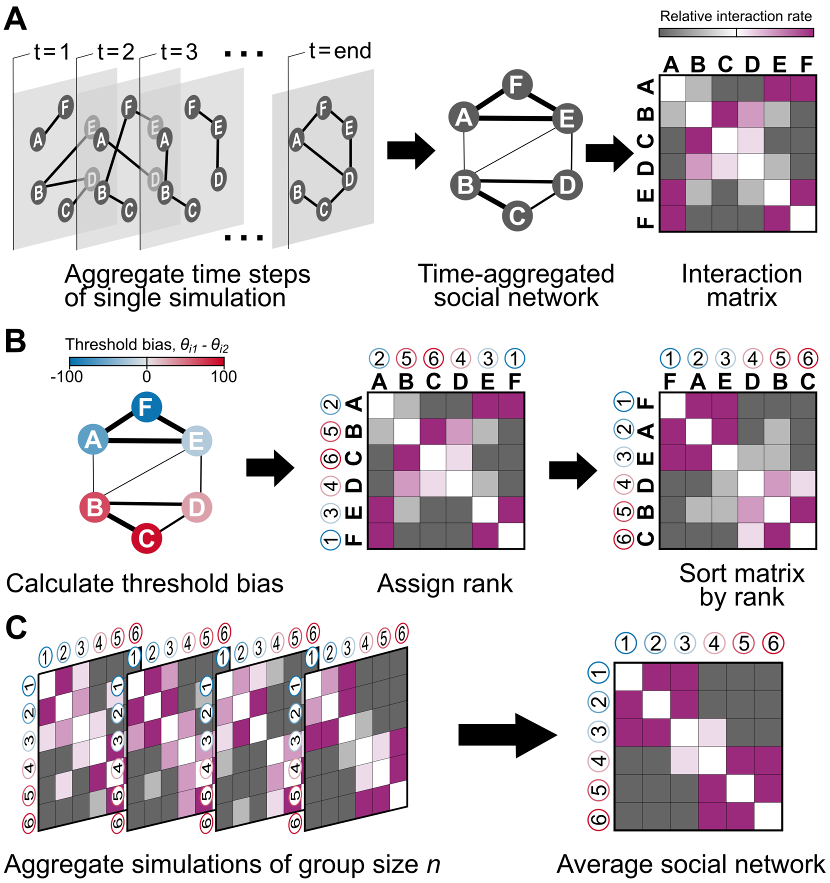

Figure S1: **Outline of methods for analysing social networks of simulated social systems.** (*A*) The social network, or interaction matrix, of a simulated social system is created at the end of a simulation by aggregating across all time points, such that edges represent the proportion of time that two individuals interacted. (*B*) The difference between an individual's thresholds at the end of the simulation is used to quantify the internal bias of each individual. Individuals are then ranked by this threshold bias, and then the rows and columns of the interaction matrix are sorted according to this ranking. (*C*) Using the sorted matrices across all replicate simulations of a given group size, an average interaction matrix can be calculated.

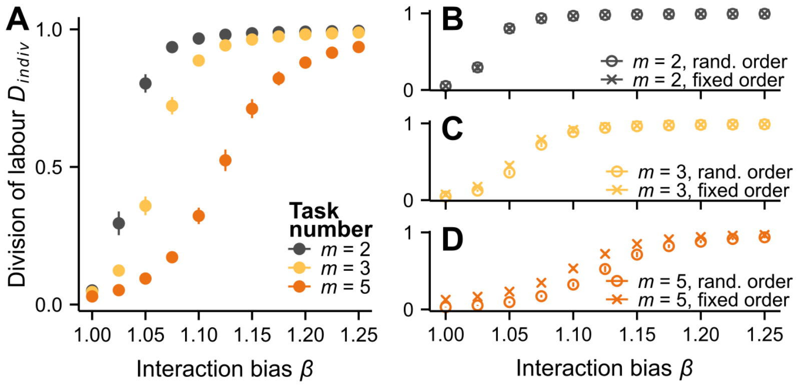

Figure S2: **The effect of altering task number and stimulus encounter order on the emergence of DOL.** (A) The pattern of emergent DOL as a function of both interaction bias β and task number m. Additional panels show the subsequent effect of fixing stimulus encounter order in the (B) two-task, (C) three-task, and (D) five-task scenario. Normally, inactive individuals encounter stimuli in a random order, but we ran simulations in which inactive individuals always encountered stimuli in sequential order, i.e., task 1, task 2, ..., task m. Points represent mean ($\pm$s.d.) of 100 replicates. See Table 1 for parameter values.

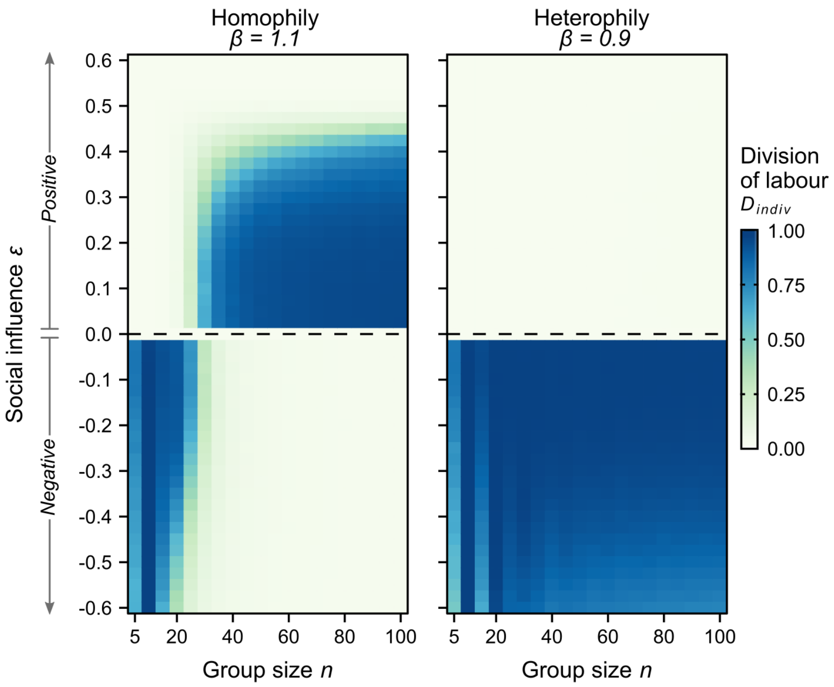

Figure S3: **Social influence parameter sweep under different conditions of interaction bias.** Colour represents the mean of 100 simulations using that parameter combination. As with the sweeps across interaction bias, we find that homophily results in scaling either group size, while heterophily does not. See Table 1 for parameter values.

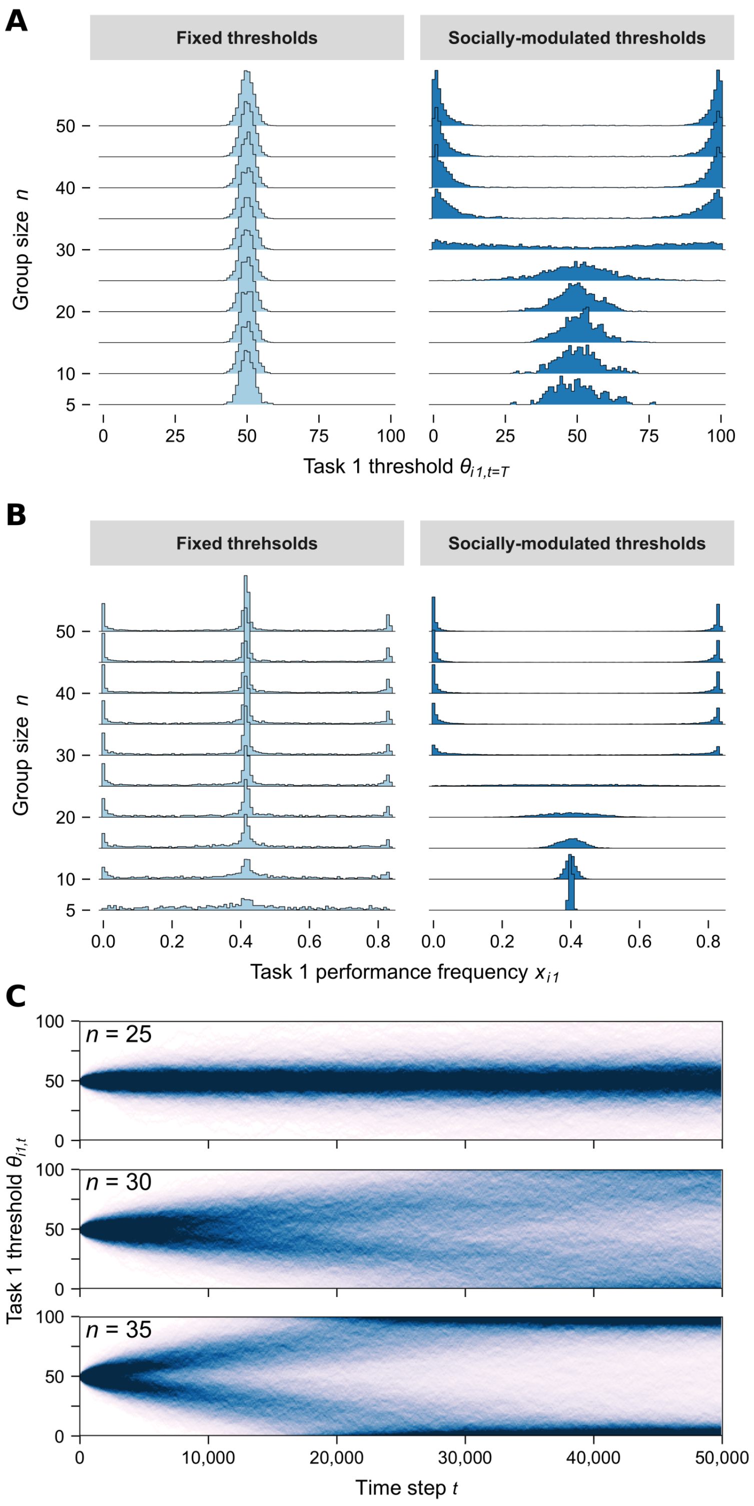

Figure S4: **Socially-modulated thresholds result in divergent thresholds and behaviour among group members.** Each plot contains all 100 replicates per group size. (*A*) By group size and model, the distribution of the final task 1 threshold value of all individuals at the end of simulations. (*B*) By group size and model, the distribution of task 1 performance frequency. (*C*) The time evolution of threshold, $\theta_{i1,t}$, in the socially-modulated threshold model across all individuals in three example group sizes demonstrating the transition from a behaviourally-homogeneous to a behaviourally-specialized society. See Table 1 for parameter values.

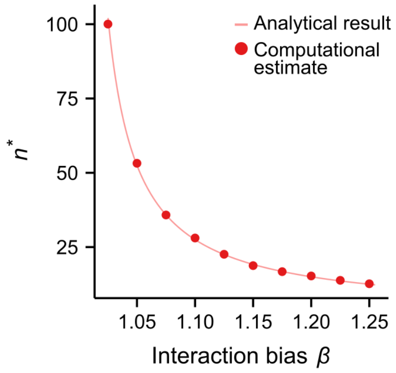

Figure S5: **The group size at which active individuals are more likely to interact with those performing the same behaviour decreases with increasing interaction bias.** Results are under conditions homophily with positive influence. The line represents the analytical result as determined by Equation (8). Points represent the estimated n^* as calculated using the computation model. The computational estimate was determined by simulating the first 6,000 time steps and comparing DOL at $t = 1,000$ and $t = 6,000$. The critical group size, n^*, at which an active individual is equally likely to interact with either behavioural type was estimated by finding the group size at which DOL did not change between these two time points. If DOL went down between these two time points, it indicated that active individuals were more likely to encounter individuals performing other tasks and therefore were becoming more similar (and therefore less specialized) over time. On the other hand, if DOL increased over time, individuals were more likely to encounter individuals performing the same task and were therefore specializing. See Table 1 for parameter values.

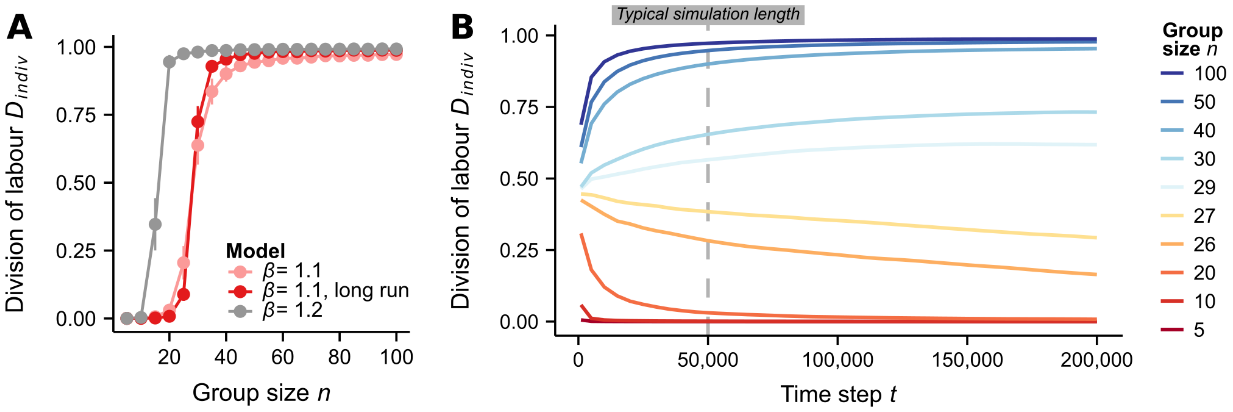

Figure S6: **Simulating longer does not qualitatively change results.** (*A*) Comparing two runs of a model with $\beta = 1.1$—one of which was run for 200,000 time steps—with a model with higher interaction bias, $\beta = 1.2$. Running models with lower interaction bias for longer results in DOL at larger group sizes that more closely resembles models run with higher interaction bias. Points represent the mean ($\pm$s.d.) of 100 replicate simulations. (*B*) DOL over time during the course of simulations for a sample of group sizes. Each line represents the average of 100 simulations. Vertical dashed line indicates the normal simulation run time presented in the main text. See Table 1 for parameter values.

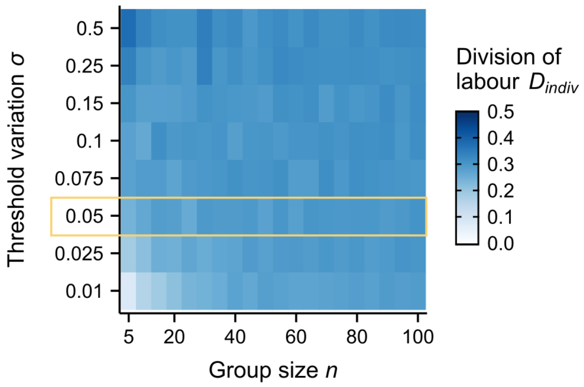

Figure S7: **The effect of initial threshold variation σ on emergent DOL in the fixed response threshold model.** Colour represents the mean of 100 simulations for each parameter combination. The gold box outlines the value used in the main text. See Table 1 for parameter values.

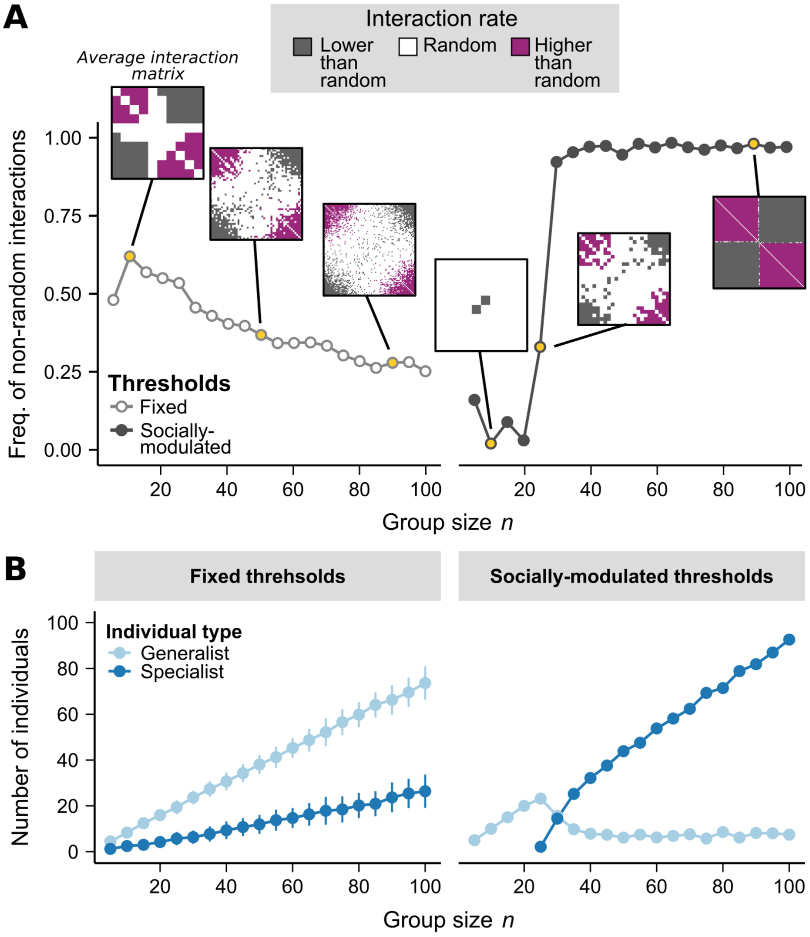

Figure S8: **Socially-modulated thresholds result in different social network scaling than fixed thresholds due to the absence of task generalists.** (*A*) The proportion of all interactions that were significantly higher or lower than the rate expected under random mixing by model type. Insets show the average interaction matrix for a corresponding group size in the model. (*B*) Average number of generalists and specialists by group size and model type. Specialists were defined as those individuals who spent > 80% of their activity on one task. Points represent the mean (±s.d.) of 100 replicate simulations. See Table 1 for parameter values.
